# Undissolved Ilmenite Mud from TiO_2_ Production—Waste or a Valuable Addition to Portland Cement Composites?

**DOI:** 10.3390/ma13163555

**Published:** 2020-08-12

**Authors:** Filip Chyliński, Jan Bobrowicz, Paweł Łukowski

**Affiliations:** 1 Instytut Techniki Budowlanej, 00-611 Warsaw, Poland; j.bobrowicz@itb.pl; 2 Faculty of Civil Engineering, Warsaw University of Technology, 00-661 Warsaw, Poland; p.lukowski@il.pw.edu.pl

**Keywords:** titanium dioxide, undissolved ilmenite mud, cement composites, addition for concrete, valorisation of waste, heat evaluation, silica fume, fly ash, trass

## Abstract

This paper presents a method of utilising ilmenite MUD created during the production of titanium dioxide (TiO_2_) according to the sulphate method as an additive for Portland cement composites. After the production process, undissolved MUD was additionally rinsed with water and filtrated in the factory to make it more useful (R-MUD) for implementation and also to turn back some of the by-products of the production of TiO_2_. R-MUD is less hazardous waste than MUD. It has a lower concentration of sulphuric acid and some heavy metals. The rinsing process raised the concentration of SiO_2_, which is a valuable part of R-MUD because of its potential pozzolanic activity. This means that the R-MUD might be a reactive substitute of part of Portland cement in building composites. The article presents the results of research on the pozzolanic activity of R-MUD and other materials with proved pozzolanic activity, such as silica fume, fly ash and natural pozzolana (trass). Tests were performed using thermal analysis techniques. The tests showed that the pozzolanic activity or R-MUD after three days is at the same level as silica fume and after 28 days it is twice as high as the activity of fly ash. Beyond the 180th day of curing, R-MUD had the same level of activity as fly ash. The summary is supplemented by calorimetric tests, which confirm the high reactivity of R-MUD compared to other commonly used concrete additives, already in the initial hydration period. In summary, heat of hydration after 72 h of Portland cement with R-MUD is at the same level as the heat of hydration of Portland cement with silica fume and also pure Portland cement grout. The results confirm that the process of formation of micro-silica contained in R-MUD react with calcium hydroxide to form the C-S-H phase, which is responsible for the microstructure of cement composites.

## 1. Introduction

**Figure 1:**
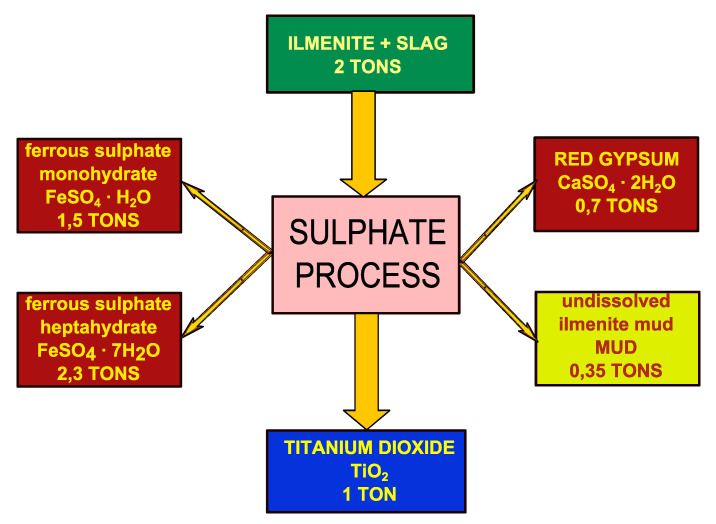
Wastes generated per tonne of titanium dioxide (TiO_2_) in the sulphate method (based on data from a factory in Huelva, Spain [5]).

Titanium dioxide (TiO_2_) is mainly produced in one of two ways: The sulphate method and the chloride method. Global world production of TiO_2_ in 2019 reached 7.2 million tonnes. The sulphate method is used to produce 45% of world production while in Europe this method takes about 70% of production [1]. The sulphate method of TiO_2_ production is difficult and generates a variety of waste from which not all can be used further (Figure 1).

Undissolved ilmenite MUD waste occurs when ilmenite is leached with sulphuric acid and insoluble residues remain. Its high sulphuric acid content (about 14%) makes ilmenite MUD a hazardous waste. The raw material used to produce TiO_2_ comes from various sources and sometimes MUD contains radioactive isotopes, which limits its safe application as a constituent of building materials. Globally, approximately 1.1 million tonnes of MUD are created each year in the production of TiO_2_ using the sulphate method [1,2,3,4,5].

Historically, this waste has been utilised in various areas of the economy, from agriculture and construction to dumping in the North Sea. According to the authors, more attention should be paid to its use in the area of construction [6,7,8,9]. One of the ways of utilising inorganic production waste is to use it as a component of cement composites, e.g., concrete [4,6,8]. Sometimes it is used as a substitute for aggregates [9,10,11] or as a substitute for parts of cement [12,13], which depends mainly on its waste-binding abilities. Waste with potential binding properties is much more valuable and has a greater application potential. This is related to the fact that it reduces the amount of cement in the binder, which gives measurable ecological and economic benefits.

The chemical, thermal and mechanical treatment of raw materials, and the resulting MUD, can create new metastable phases, which can be reactive in cement composites. The raw materials used for the production of titanium dioxide by the sulphate method contain large quantities of silicone dioxide, which can be reactive after activation by increasing the specific surface area and treatment with sulphuric acid at elevated temperatures. Examples of this type of activation, which leads to an increase in the activity of additives recorded in calorimetric studies [14] are modified fly-ash [15] and blast furnace slag, which are among the most popular concrete additives. One of the methods for their activation consists in additional grinding, which results in a significant increase in specific surface area [11,16,17,18,19,20].

According to EN 206, [21] concrete additives are classified in two groups: Type I and Type II. Type I additives are almost inert materials, which are used as concrete fillers. Type II additives are materials with hidden hydraulic and/or pozzolanic properties, used as a substitute for cement in concrete.

Ilmenite MUD, due to its high content of silica, which was subjected to intensive mechanical which increased highly its specific area and also chemical treatment, might be a highly active pozzolanic material. The treatment that accompanies the process of waste formation and the constitution of this material may suggest that the composition contains nano-particles of SiO_2_ and TiO_2_ [22,23], which, as shown in the literature, are characterised by high reactivity and influence the microstructure of composites [24,25,26,27,28]. The nano particles of TiO_2_ might also have a photocatalytic properties [27]. The aim of the rinsing and filtration process was to receive more of the titanyl sulphate from the MUD, which is a valuable substitute for TiO_2_ production, and to decrease the concentration of sulphate acid and some of the heavy metals.

Rinsing is one of the ways to remove harmful constituents from waste [28]. Ilmenite MUD, after rinsing and filtration, which significantly reduces the content of sulphuric acid, can be used as a component of concrete. The filtrate is rich in useful by-products, and can also be returned to the TiO_2_ production process.

The aim of this article is to show that waste material, such as R-MUD which is classified as hazardous [29], might be valuable raw material for the production of cement composites and may also allow for the replacement of part of the cement in the binder [30]. This is a sustainable action to limit the use of natural raw materials while taking advantage of the potential opportunities offered by waste in landfills. The results presented in this article are part of a programme aimed at evaluating the possibility of using R-MUD as a safe and valuable reactive additive to concrete [4]. The research programme presented in this paper focused mainly on the pozzolanic reactivity of R-MUD caused most probably by the presence of reactive silicon dioxide in the waste. Other tests were also performed to compare the composition of waste with literature data and to search for potential threats to the durability of cement composites.

Pozzolanic activity of material might be determined by several methods which can be classified as direct and indirect. Direct methods are focused on measuring the changes of concentration of Ca(OH)_2_ using DTA, XRD or classical chemical titration [31]. An example of such test is Frattini method in which the pozzolana is mixed witch CEM I Portland cement with the addition of water [32]. There is also a simplified modification of Frattini test—saturated lime method in which the saturated solution of calcium hydroxide is used instead of Portland cement.

The indirect methods are used to determine the activity index by comparing physical properties of cement composites with and without the addition of pozzolana. The physical property in most cases is compressive strength of cement mortars. This method is also used in European standards to determine the activity of fly ash or silica fume [33,34].

## 2. Materials and Methods

### 2.1. Materials

The MUD used in this study came from a European TiO_2_ factory, which uses the sulphate method in its production process. The raw material used in the production of TiO_2_ is a mixture of ilmenite and ilmenite slag. The ilmenite came from a deposit near Tellnes, Norway. The content and morphology of this deposit is well known and described widely in the literature [35,36]. It contains mainly hemoilmenite, orthopyroxene, plagioclase and also biotyte, magnetite, olivine, apatite, Fe-Ni-Co-Cu sulphates and other minerals. Ilmenite slag came from electro smelting in a Norwegian factory used as feedstock of the same ilmenite mentioned above. The enrichment of the ore is based on the electro smelting process and the reduction of part of iron from the hemoilmenite and magnetite casting process [37,38]. In this paper, research based on rinsed ilmenite MUD (R-MUD) from a European factory is described. The MUD was rinsed with water and filtrated in the factory prior to its delivery to the laboratory.

Samples of R-MUD were dried at 105 °C until they reached a constant weight and sieved using a 0.5 mm sieve before analysis. The water content in R-MUD was 25.3% by mass. Prior to the instrumental analysis (such as X-ray fluorescence (XRF), trace mercury analysis (TMA), X-ray diffraction (XRD) and differential thermal analysis (DTA)), the samples were additionally ground to reach a grain size under 0.063 mm. This paper also presents some other results based on different MUD batches other than the rinsed, received from the producer. Additional pozzolan additives used in the study are standard additives used in the concrete industry. Some have been described in detail by the authors in a previous paper [4,39,40] and are presented in Table 1.

### 2.2. Methods

#### 2.2.1. Sulphuric Acid Equivalent, Acidity Number and Sulphate Content

Sulphuric acid content, acidity number and sulphate content in R-MUD were measured to check the effect of the rinsing process and to choose a method of neutralisation of residue acid.

The sulphuric acid equivalent was measured by using the titration method with 0.1 M NaOH. The acidity number was calculated from the results of the study described above. Sulphate content was measured according to the EN 196-2 standard [9,41].

#### 2.2.2. Major and Trace Constituents

The major elements were measured by XRF with a Philips PW 2400 system (Philips Industrial Electronics, Almelo, Netherlands). The samples were melted with lithium tetraborate and lithium iodine to receive homogeneous glass ready for measurement.

The trace elements were also measured by using the XRF method. The samples, however, were not melted but compressed without the addition of any other substances.

The mercury concentration was measured with single-purpose atomic absorption spectrometer TMA AMA 254 Altem system (Leco Corporation, Shoreham, MI, USA). 

#### 2.2.3. XRD

The mineralogical study was performed by an X-ray diffraction system VEB Freiberger Pragisionsmechanik TUR-M-62 system (Freiberg Instruments GmbH, Freiberg, Germany) with Kα radiation of Cu excited by 20 mA of intensity and 40 kV of tension. The mineralogical identification was carried out using the X-rayan software (ver. 4.0.1, Koma, Warsaw, Poland). 

#### 2.2.4. Scanning Electron Microscopy (SEM) with X-ray Microanalysis

Structure observations were made using a scanning electron microscope (SEM) produced by Zeiss, model Sigma 500 VP (Carl Zeiss Microscopy GmbH, Köln, Germany). Secondary electron (SE) and backscattered electron (BSE) images were collected. Phase compositions and mapping were analysed using the EDS detector model Oxford Ultim Max 40 (Oxford Instruments, High Wycombe, UK). 

#### 2.2.5. Thermal Analysis—Pozzolanic Activity

Pozzolanic activity was measured using differential thermal analysis (DTA). The samples were prepared by mixing R-MUD with Ca(OH)_2_ (analytical grade) and demineralised water (mass ratio 1:1:2) [42,43,44], and then stored in sealed containers in temperature 20 ± 2 °C. After 1 to 360 days, the hydration process by adding the ethanol (99.8%) in the amount of about five times larger than the volume of sample and put under the vacuum (30 kPa) for two hours. Then, samples were dried in an oven at a temperature of 40 °C for 24 h. Next, they were ground and sieved using a 0.063 mm sieve. The DTA tests were carried out using Thermal Analysis TA Instruments system SDT Q600 (TA Instruments, New Castle, DE, USA). Tests ran in air gas flow 100 ml/min. DTA (differential thermal analysis), TG (thermogravimetry) and DTG (derivative thermogravimetry) curves were recorded in a range of temperatures from 20 to 1000 °C. The heating rate was 10 °C/min. A platinum crucible was used. The sample weight was about 20 mg. The experiment was repeated three times for each mixture and the average values were calculated.

The aim of this study was to describe a quantity of portlandite that the R-MUD was able to bind to the C-S-H phase. The quantities of portlandite and calcite were measured in each sample. The calculations also included the quantity of residue sulphuric acid in R-MUD, which bound some of the calcium hydroxide.

The tests of other materials with proven pozzolanic activities (such as fly ash, silica fume and natural pozzolana-trass) were performed in the same way. The loss on ignition of raw fly ash was also included in the calculations.

#### 2.2.6. Reactive Silicone Dioxide

Reactive silicone dioxide content was measured according to EN 196-2 and EN 450-1 [33,41]. These methods are dedicated to fly ash tests and are not validated for such materials as R-MUD. There were three measurements of this value for Fly ash and R-MUD.

#### 2.2.7. Calorimetric Tests

The process of testing the amount and rate of heat release was carried out on cement pastes, the composition of which was determined on the basis of the results of own research described elsewhere [6]. As described in [6], the most interesting results were obtained for 10 divided by 20% of R-MUD content added to CEM I 42.5R. It was established that the optimum amount of R-MUD added to cement was about 15% of CEM I 42.5R. Comparisons of the reactivity of other examined pozzolanic admixtures were also carried out on samples of mixtures of 15% addition to cement. The test procedure was as follows:

- 8 g samples *(S)* of cement and appropriate mixtures of cement and pozzolana were placed in special calorimetric vessels;
- 4 g of water *(W)* were placed in syringes;
- after thermostatting was finished, the measurement was started and water was introduced through injection needles into the measurement chambers.

The calorimetric tests were carried out at a temperature of 20 °C in a special measurement system on an isothermal calorimeter [45] for a minimum of 72 h.

The experiment was repeated three times for each mixture and the average value was calculated.

## 3. Results and Discussion

### 3.1. Acid and Sulphate Content

The sulphuric acid equivalent in dry R-MUD was 1.23%. This value was much lower than the value measured for MUD (about 14%). The values of acid were 17 and 193 mg_KOH_/g respectively.

Sulphate content (as SO_3_) in dry R-MUD was 1.0%. This level of sulphate concentration does not exclude the potential use of R-MUD as a concrete addition. As an example, in EN 450-1 [33] the limit of SO_3_ in fly ash for concrete is 3.0%.

In order to increase the pH value of R-MUD water solution to about seven, the addition of about 1% of calcium hydroxide is suitable. As a product of the hydration reaction of Portland cement, about 20% of calcium hydroxide is obtained. This means that further neutralization of R-MUD might not be necessary.

### 3.2. Major and Trace Elements (XRF and TMA)

Table 2 and Table 3 present the content of major and trace elements in MUD and R-MUD. The main constituents of MUD and R-MUD were silicon, titanium and iron compounds. Compared to the literature data [5,8], the rinsing process increased the concentration of silicon dioxide and titanium, while simultaneously decreasing the iron content. Furthermore, the proportions of trace elements were modified—the concentrations of some trace elements were increased (chromium and strontium), while others were decreased (copper and nickel).

Heavy metals presented in R-MUD are effectively immobilised in the cement matrix [46,47,48,49,50].

### 3.3. Mineral Composition

**Figure 2:**
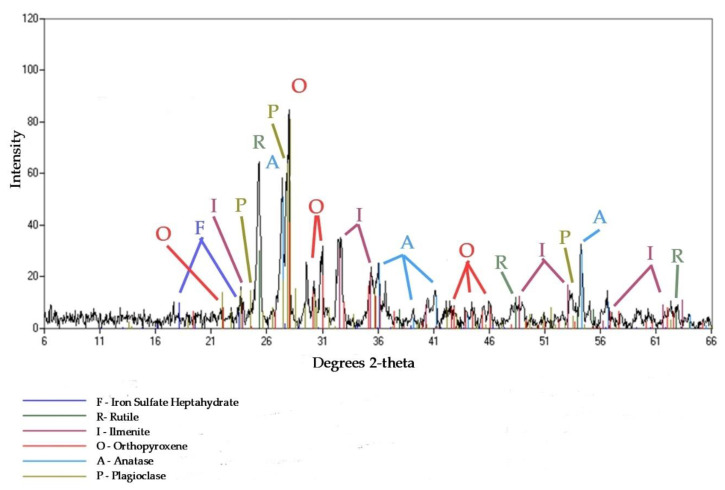
XRD pattern of R-MUD sample.

The mineral composition determined by XRD (Figure 2) shows the following major species in R-MUD: Anatase, rutile, ilmenite, orthopyroxene and plagioclase. There were also some minor species, like zirconium and quartz. The presence of iron sulphate heptahydrate was not ruled out. The concentration of magnesium in cement composites is limited because of the expansive character of some of its compounds. The results of XRD tests show that the magnesium ions were mostly bonded in orthopyroxenes and silicates, which do not decrease the durability of the cement matrix.

### 3.4. Scanning Electron Microscopy (SEM)

**Figure 3:**
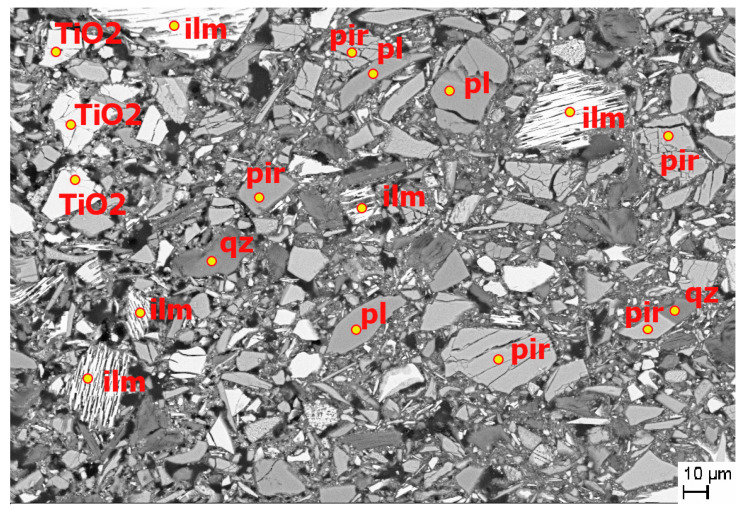
Backscattered electron (BSE) image of R-MUD sample (backscattered electron mode). Identified constituents: Pir—orthopyroxene, ilm—ilmenite, qz—quartz, pl—plagioclase and TiO_2_—titanium dioxide.

**Figure 4:**
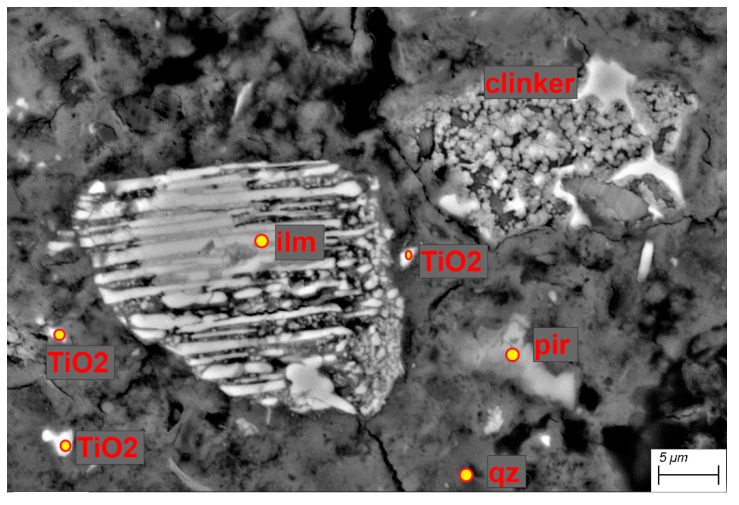
BSE image of cement mortar containing R-MUD; pir—orthopyroxene, ilm—ilmenite, qz—quartz and TiO_2_—titanium dioxide.

**Figure 5:**
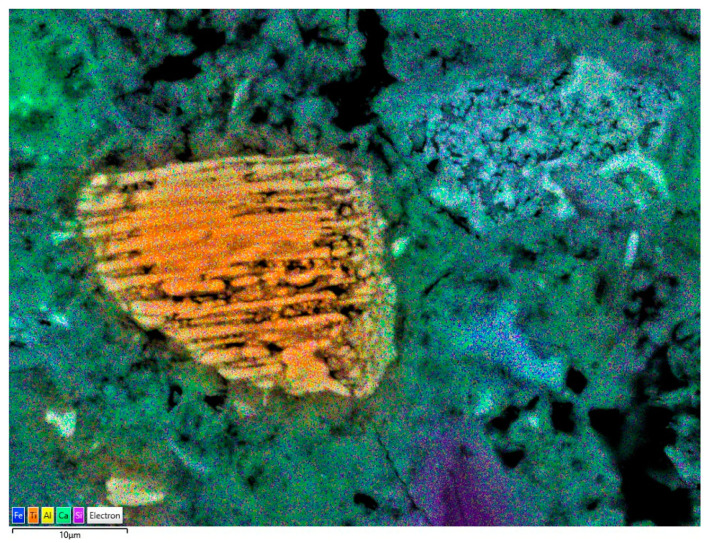
Energy dispersive X-ray spectroscopy (EDS) mapping of area presented in Figure 4.

BSE (backscattered electron mode) observations were carried out for the R-MUD samples (Figure 3) and for the CEM I cement mortar containing R-MUD (Figure 4 and Figure 5). The waste contained insignificantly modified grains of plagioclase and orthopyroxene and highly leeched deposit minerals with cracks and defects. A big part of the R-MUD took the form of amorphous gel of various silicate-titanium-iron-sulphate proportions.

Figure 4 presents the mortar with R-MUD particles surrounded by the C-S-H phase and the grain of clinker. The particles of R-MUD are active constituents and their ions are a part of C-S-H gel what might be observed in Figure 5 by the energy dispersive X-ray spectroscopy (EDS) mapping as an orange hallo surrounding ilmenite and TiO_2_ grains, and also by the violet halo surrounding quarts grain. Composition of C-S-H gel near the clinker grain is typical as for Portland cement but closer to the ilmenite and TiO_2_ grains the composition of C-S-H gel is modified by the titanium ions.

### 3.5. Pozzolanic Activity (DTA)

**Figure 6:**
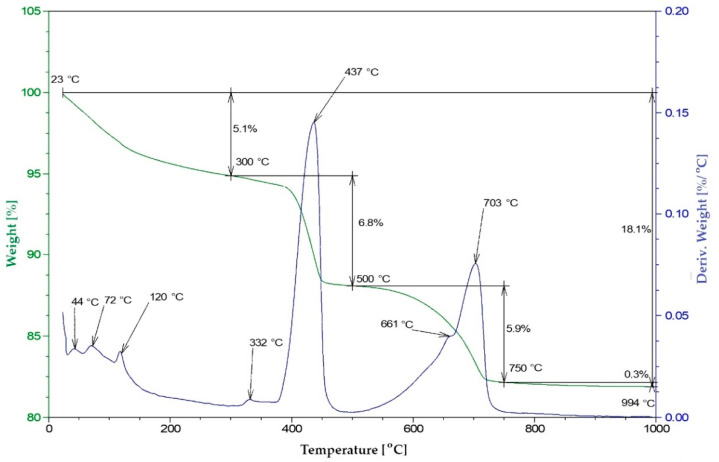
TG and differential thermal analysis (DTA) thermogram of R-MUD/Ca(OH)_2_ samples after 7 days of curing.

Figure 6 shows the thermogram curve of the R-MUD/Ca(OH)_2_ mixture after 7 days of curing. In the range of temperature 20–300 °C, the thermal effects associated with the evaporation of unbounded water and the decomposition of the C-S-H phase [51] were observed. In the range of 300–500 °C, the endothermic effect of the dehydration of calcium hydroxide was noticed with a maximum effect at 437 °C. At temperatures 600–750 °C, the process of decomposition of calcium carbonate was observed with a maximum endothermic effect at 703 °C. The presence of calcium carbonate in the samples was probably the result of drying of the samples in an oven in air prior to analysis. The content of calcium carbonate in all samples was at the same level.

**Figure 7:**
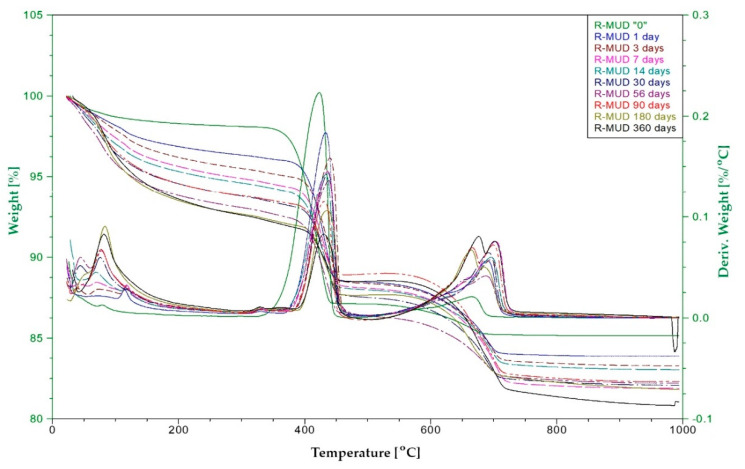
TG and DTA thermogram of R-MUD/Ca(OH)_2_ samples after different periods of curing.

**Figure 8:**
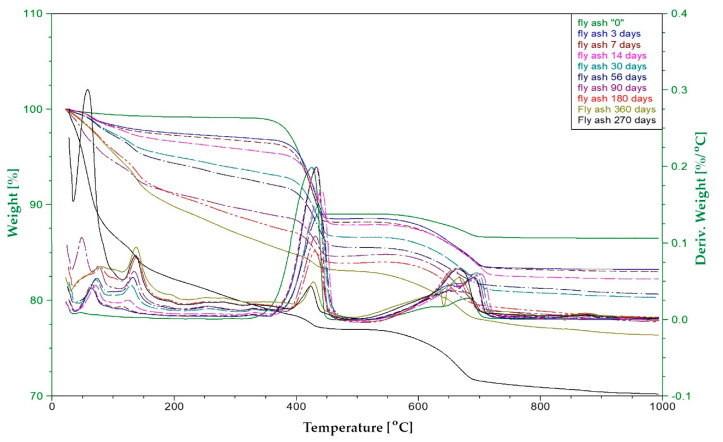
TG and DTA thermogram of fly ash/Ca(OH)_2_ samples after different periods of curing.

**Figure 9:**
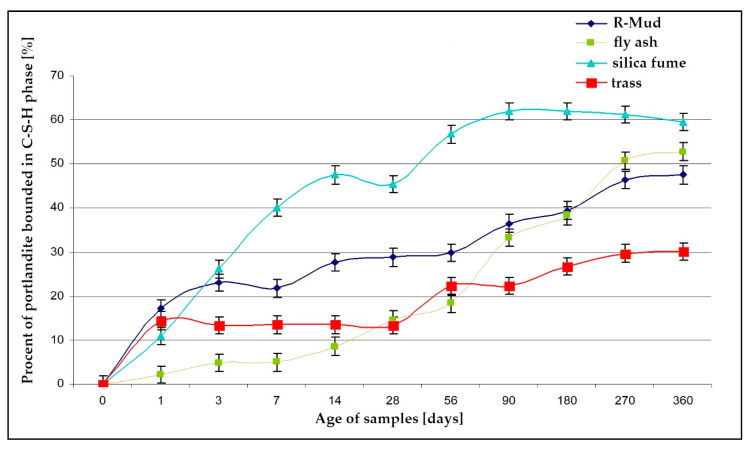
Pozzolanic activity of different materials as a percentage of conversion of portlandite into C-S-H phase.

Figure 6 and Figure 7 present TG and DTA curves of the R-MUD/Ca(OH)_2_ mixture and fly ash/Ca(OH)_2_ mixture (Figure 8) as examples of material with pozzolanic activity. The progress of the reaction was calculated by measuring the differences between the concentration of calcium hydroxide in each sample and the reference sample, which was tested just before mixing. Figure 9 presents pozzolanic activity as a percentage of calcium hydroxide bound to the C-S-H phase compared to other materials. The percentage of conversion for R-MUD was larger than for the fly ash and trass, especially in the first 90 days of curing. After 180 days, the R-MUD samples were able to convert about 40% of portlandite mass into the C-S-H phase. The effectiveness of the pozzolanic reaction for R-MUD in the first 56 days of curing was about twice as big as for the fly ash samples.

### 3.6. Reactive Silicon Dioxide

The contents of reactive silicon dioxide in R-MUD and fly ash reach 10% and 38%, respectively. Even though the method of analysis was not validated for R-MUD, the effectiveness of the pozzolanic activity (Figure 9) of this material was surprisingly high. This may be due to the larger specific surface area of R-MUD waste compared to fly ash or trass, or the chemical treatment of R-MUD during the production of TiO_2_. It can also not be excluded that nano-TiO_2_ will react with calcium hydroxide. In this series of studies, no attempt was made to determine the content of nano-TiO_2_; however, it cannot be excluded that this type of phase occurs here. High reactivity of R-MUD was also confirmed by calorimetric studies. The main reason of this result might be the stoichiometry of the formed C-S-H phase in which the CaO/SiO_2_ ratio is higher.

### 3.7. Calorimetric Test Results

Calorimetric studies were carried out to confirm the reactivity of R-MUD pozzolanic used as an admixture to Portland cement in comparison with admixtures of known and confirmed activity also described above [51,52,53,54].

**Figure 10:**
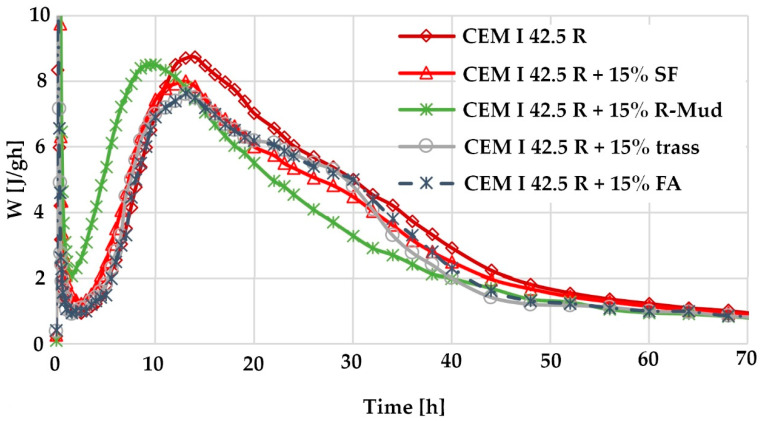
Rate of heat development during hydration of cement of tested mixtures of cement and individual pozzolana at a temperature of 20 °C at w/s = 0.5 − W = dQ/dt (J/g h).

The results of calorimetric tests are shown in Figure 10. This figure shows high activity of the CEM I 42.5R mixture containing 15% R-MUD already in the first hours of hydration. High reactivity of this admixture may also, in part, result from the phase transition taking place in a strongly alkaline environment, especially at the very beginning of the recorded thermal effects. Such a conclusion is drawn from the analysis of Ramakokovhu et al. [55], who claim that R-MUD components may undergo phase transformations with exothermic effects, which they observed while conducting studies at elevated temperatures. In calorimetry, the studies are carried out at a constant temperature of 20 °C but, here, such phase transformations may occur under the influence of a strongly alkaline environment.

The results shown in Figure 10 and Table 4 illustrate the practically comparable reactivity of R-MUD with the well tested and very active admixture of silica fume [56,57,58,59]. This can be seen even if we try to estimate the effect of the initially very high reactivity, because during the first half hour more than twice as much heat is released from the cement and R-MUD mixture than from the cement and silica fume mixture. During this period, the heat released and the accompanying reactions should not affect the microstructure of the binder. Most probably, the heat emitted at the very beginning of contact of the mixture containing R-MUD with water is mainly associated with dissolution and neutralisation effects and possibly the mentioned phase transformations occurring [55] as a result of the mixture’s contact with a strongly alkaline paste environment. Comparison of the heat released after 24 h of hydration of R-MUD and other additives including silica fume still indicates a very high reactivity of this additive, which is at the level of 18%—an advantage of R-MUD over SF (silica fume). The advantage of the amount of heat released decreases with time, and practically shows a difference of only 1.4 J/g or 0.6% after 3 days of reaction. The results obtained in the tests carried out with the isothermal calorimeter perfectly correspond to the transformations associated with the study of the initial reactivity of the examined pozzolanic admixtures with calcium hydroxide by thermogravimetry (Figure 9).

## 4. Conclusions

The results of the research and the analysis presented above allow us to conclude that:

▪ the correction of the sulphuric acid content and the washing out of some of the soluble salts allows to obtain a new quality of waste—R-MUD, which is less hazardous than MUD and allows to consider other possibilities of application. Moreover, the resulting filtrate is a valuable raw material for further production of TiO_2_;
▪ it is justified to expect that minimising the sulphuric acid content in R-MUD to a level below 1% will not threaten the durability of cement composites [4];
▪ cement composites allow to immobilise heavy metals, which are found in R-MUD [50]. Attempts to utilise waste containing heavy metals and to protect it against leaching from cement composite materials have been described in the literature many times [5,48,49,50];
▪ R-MUD has a relatively low content of silicon dioxide (about 35%) and reactive silicon dioxide (about 10%) with surprisingly high pozzolanic activity. This allows for the effective use of this material as a reactive admixture to the concrete substitute part of the cement;
▪ R-MUD most likely gains in reactivity as a result of aggressive chemical, thermal and mechanical treatment and may contain nano-silica and nano-TiO_2_ [20]. The latter conclusion, however, needs to be confirmed by further studies;
▪ the results of calorimetric and thermogravimetric tests confirm the high activity of R-MUD and the ability to form the C-S-H phase responsible for the microstructure of cement grouts.

In answer to the question stated in the title of the article (i.e., whether undissolved ilmenite mud from TiO_2_ production is a waste or a valuable addition to Portland cement composites), the results presented in this article show that this material should be treated as a potential source of valuable raw material for sustainable concrete production.

In further studies on R-MUD the authors are planning to investigate the rate of hydration of Portland cement with addition of various amount of R-MUD and also the influence of waste on hydration of different types of cement containing fly ash or granulated blast furnace.

## Figures and Tables

**Table 1:** Concentration [%] of major elements in samples and relevant surface.

Elements	CEM I 42.5R	R-MUD	Silica Fume	Trass	Fly Ash
SiO_2_	20.60	35.07	91.40	77.05	51.51
CaO	64.41	3.09	0.09	0.30	3.82
TiO_2_	–	33.05	<0.01	0.09	1.09
Al_2_O_3_	4.13	5.53	0.15	12.35	25.71
Fe_2_O_3_	3.38	9.65	0.65	0.07	8.51
MgO	0.89	7.26	1.51	0.06	2.53
Na_2_O	0.24	1.10	1.02	3.25	1.37
K_2_O	0.56	0.26	1.07	4.85	2.73
MnO	–	0.53	0.06	0.01	0.10
P_2_O_5_	–	0.01	0.13	0.02	0.31
(SO_3_)	2.97	0.05	0.01	<0.01	0.48
(Cl)	0.070	0.017	0.115	0.011	0.015
Specific surface area ^1^ [cm^2^/g]	4060	8390	23,160	6030	4020

^1^ Determined by Blane method except for the silica fume which was measured by BET method (Brunauer-Emmett-Teller surface area analysis).

**Table 2:** Concentration (%) of major elements in sample of waste before (MUD) and after rinsing (R-MUD) (Data from reference [4]).

Element	SiO_2_	TiO_2_	Fe_2_O_3_	MgO	Al_2_O_3_	CaO	Na_2_O	MnO	K_2_O	P_2_O_5_
MUD	23.56	27.20	16.65	5.39	4.08	2.49	0.82	0.33	0.13	0.01
R-MUD	35.07	33.05	9.65	7.26	5.53	3.09	1.10	0.53	0.26	0.01

**Table 3:** Concentration (ppm) of trace elements in sample of waste before (MUD) and after rinsing (R-MUD).

**Element**	**As**	**Br**	**Ce**	**Co**	**Cr**	**Cu**	**Ga**	**Hf**	**La**	**Mo**	**Nb**	**Ni**	**Pb**
MUD	18	4	21	77	253	313	11	19	29	3	57	194	<3
R-MUD	22	5	38	79	294	201	7	24	38	4	137	151	<3
**Element**	**Rb**	**Sr**	**Bi**	**Th**	**U**	**V**	**Y**	**Zn**	**Zr**	**Cd**	**Sn**	**Hg**	
MUD	3	155	4	6	7	558	11	519	1065	10	10	0.014	
R-MUD	11	220	3	28	5	795	19	504	1296	11	<2	0.025	

**Table 4:** Comparison of hydration heat released from CEM I 42.5R and cement with 15% addition of the tested pozzolana.

Time [h]	Total Heat of Hydration [J/g]				
	CEM I 42.5R	CEM I 42.5R + 15% Trass	CEM I 42.5 R + 15% Silica Fume	CEM I 42.5R + 15% Fly Ash	CEM I 42.5R + 15% R-MUD
0.5	6.6	1.4	7.0	3.9	15.5
1	7.8	1.7	8.4	5.0	17.4
2	9.2	3.1	9.9	6.1	19.6
4	13.0	5.5	12.7	8.3	25.9
6	20.1	8.9	17.7	11.8	36.9
12	62.6	43.5	55.8	43.8	85.5
18	110.0	88.8	100.1	89.8	127.9
24	147.2	125.7	135.6	126.2	159.5
36	196.2	178.3	188.2	179.4	199.8
48	221.8	203.1	215.5	204.0	221.4
60	237.1	218.2	231.9	218.9	235.0
72	248.6	228.9	243.7	229.5	245.1

## References

[1] Titanium Dioxide (TiO_2_) Market 2019 Global Industry Analysis By Key Players, Share, Revenue, Trends, Organizations Size, Growth, Opportunities, and Regional Forecast to 2025. https://www.grandviewresearch.com/industry-analysis/titanium-dioxide-industry.

[2] Contreras M., Martín M.I., Gázquez M.J., Romero M., Bolívar J.P. (2014). Valorisation of ilmenite mud waste in the manufacture of commercial ceramic. Constr. Build. Mater..

[3] Contreras M., Teixeira S.R., Lucas M.C., Lima L.C., Cardoso D.S., Da Silva G.A., Gregório G.C., De Souza A.E., Dos Santos A. (2016). Recycling of construction and demolition waste for producing new construction material (Brazil case-study). Constr. Build. Mater..

[4] Chyliński F., Kuczyński K., Łukowski P. (2020). Application of ilmenite mud waste as an addition to concrete. Materials.

[5] Gázquez M.J., Mantero J., Bolívar J.P., García-Tenorio R., Vaca F., Lozano R.L. (2011). Physico-chemical and radioactive characterization of TiO_2_ undissolved mud for its valorization. J. Hazard. Mater..

[6] Bobrowicz J., Chyliński F. (2016). The influence of ilmenite mud waste on the hydration process of Portland cement. J. Therm. Anal. Calorim..

[7] Contreras M., Martín M.I., Gázquez M.J., Romero M., Bolívar J.P. (2016). Manufacture of ceramic bodies by using a mud waste from the TiO_2_ pigment industry. Key Eng. Mater..

[8] Llanes M.C., González M.J.G., Moreno S.M.P., Raya J.P.B. (2018). Recovery of ilmenite mud as an additive in commercial Portland cements. Environ. Sci. Pollut. Res..

[9] Harasymiuk J., Rudziński A. (2020). Old dumped fly ash as a sand replacement in cement composites. Buildings.

[10] Fan C.C., Huang R., Hwang H., Chao S.J. (2015). The effects of different fine recycled concrete aggregates on the properties of Mortar. Materials.

[11] Chung S.Y., Elrahman M.A., Sikora P., Rucinska T., Horszczaruk E., Stephan D. (2017). Evaluation of the effects of crushed and expanded waste glass aggregates on the material properties of lightweight concrete using image-based approaches. Materials.

[12] Mohamed O. (2018). Durability and compressive strength of high cement replacement ratio self-consolidating concrete. Buildings.

[13] Konkol J. (2019). Fracture Toughness and fracture surface morphology of concretes modified with selected additives of pozzolanic properties. Buildings.

[14] Moghaddam F., Sirivivatnanon V., Vessalas K. (2019). The effect of fly ash fineness on heat of hydration, microstructure, flow and compressive strength of blended cement pastes. Case Stud. Constr. Mater..

[15] Karthiyaini S. (2016). Physicochemical propertiesof alkali activated fly ash based geopolymer concrete: A review. Int. J. Earth Sci. Eng..

[16] Czarnecki L., Kapron M. (2010). Sustainable construction as a research area. Int. J. Soc. Mater. Eng. Resour..

[17] Marthong C., Agrawal T.P. (2012). Effect of fly ash additive on concrete properties. Int. J. Eng. Res. Appl..

[18] Giergiczny Z. (2019). Fly ash and slag. Cem. Concr. Res..

[19] Michalik A., Babińska J., Chyliński F., Piekarczuk A. (2019). Ammonia in fly ashes from flue gas denitrification process and its impact on the properties of cement composites. Buildings.

[20] Sikora P., Augustyniak A., Cendrowski K., Horszczaruk E., Rucinska T., Nawrotek P., Mijowska E. (2016). Characterization of mechanical and bactericidal properties of cement mortars containingwaste glass aggregate and nanomaterials. Materials.

[21] EN 206+A1:2016-12 (2016). Concrete-Specification, Performance, Production and Conformity.

[22] Horoszczaruk E. (2018). Role of nanosilica in the formation of the properties of cement composites, state of the art. Cem. Wapno Bet..

[23] D’Alessandro A., Materazzi A.L., Ubertini F., Jenny S. (2020). Nanotechnology in Cement-Based Construction.

[24] Czarnecki L., Schorn H. (2014). Nanomonitoring of polymer cement concrete microstructure. Restor. Build. Monum..

[25] Kurdowski W. (2014). Cement and Concrete Chemistry.

[26] Bautista-Gutierrez K.P., Herrera-May A.L., Santamaría-López J.M., Honorato-Moreno A., Zamora-Castro S.A. (2019). Recent progress in nanomaterials for modern concrete infrastructure: Advantages and challenges. Materials.

[27] Xiong J., Liang Y., Cheng H., Guo S., Jiao C. (2020). Preparation and photocatalytic properties of a bagasse. Materials.

[28] Ferone C., Colangelo F., Messina F., Santoro L., Cioffi R. (2013). Recycling of pre-washed municipal solid waste incinerator fly ash in the manufacturing of low temperature setting geopolymer materials. Materials.

[29] Wahlström M., Laine-Ylijoki J., Wik O., Oberender A., Hjelmar O. (2016). Hazardous Waste Classification.

[30] Tavakoli D., Hashempour M., Heidari A. (2018). Use of waste materials in concrete: A review. Pertanika J. Sci. Technol..

[31] Donatello S., Tyrer M., Cheeseman C.R. (2010). Comparison of test methods to assess pozzolanic activity. Cem. Concr. Compos..

[32] Kramar S., Ducman V. (2018). Evaluation of ash pozzolanic activity by means of the strength activity index test, frattini test and DTA/TG analysis. Teh. Vjesn..

[33] EN 450-1:2012 (2012). Fly Ash for Concrete-Part. 1: Definition, Specifications and Conformity Criteria.

[34] EN 13263-1+A1:2009 (2009). Silica Fume for Concrete-Part. 1: Definitions, Requirements and Conformity Criteria.

[35] Klepka M., Lawniczak-Jablonska K., Jablonski M., Wolska A., Minikayev R., Paszkowicz W., Przepiera A., Spolnik Z., Van Grieken R. (2005). Combined XRD, EPMA and X-ray absorption study of mineral ilmenite used in pigments production. J. Alloys Compd..

[36] Diot H., Bolle O., Lambert J.-M., Launeau P., Duchesne J.-C. (2003). The tellnes ilmenite deposit (Rogaland, South Norway): Magnetic and petrofabric evidence for emplacement of a Ti-enriched noritic crystal mush in a fracture zone. J. Struct. Geol..

[37] Sunde M. (2012). Organic Binder as a Substitute for Bentonite in Ilmenite Pelletization. Master’s Thesis.

[38] Samal S., Mohapatra B.K., Mukherjee P.S., Chatterjee S.K. (2009). Integrated XRD, EPMA and XRF study of ilmenite and titania slag used in pigment production. J. Alloys Compd..

[39] Bobrowicz J., Chyliński F. (2020). Comparison of pozzolanic activity of ilmenite MUD waste to other pozzolans used as an additive for concrete production. J. Therm. Anal. Calorim..

[40] Chyliński F., Kuczyński K. (2020). Ilmenite mud waste as an additive for frost resistance in sustainable concrete. Materials.

[41] EN 196-2:2013-11 (2013). Method of Testing Cement-Part. 2: Chemical Analysis of Cement.

[42] Roszczynialski W., Stępień W., Tkaczewska P., Roszczynialski E. (2014). The reliability of pozzolanic activity assessment methods in testing different fly ashes. Cem. Wapno Bet..

[43] Mertens G., Snellings R., van Balen K., Bicer-Simsir B., Verlooy P., Elsen J. (2009). Pozzolanic reactions of common natural zeolites with lime and parameters affecting their reactivity. Cem. Concr. Res..

[44] Broekmans M.A.T.M., Pöllmann H. (2018). Applied Mineralogy of Cement & Concrete.

[45] Witakowski P., Czamarska D., Bobrowicz J. (1991). Skoputeryzowany układ do pomiarów kalorymetrycznych Część I Aparatura. C Ement. Waspno. Gips..

[46] Nocuń-Wczelik W., Małolepszy J. (1995). Application of calorimetry in studies of the immobilization of heavy metals in cementitious materials. Thermochim. Acta.

[47] Giergiczny Z., Król A. (2008). Immobilization of heavy metals (Pb, Cu, Cr, Zn, Cd, Mn) in the mineral additions containing concrete composites. J. Hazard. Mater..

[48] Chen Q.Y., Tyrer M., Hills C.D., Yang X.M., Carey P. (2009). Immobilisation of heavy metal in cement-based solidification/stabilisation: A review. Waste Manag..

[49] Habib M.A., Bahadur N.M., Mahmood A.J., Islam M.A. (2012). Immobilization of heavy metals in cementitious matrices. J. Saudi Chem. Soc..

[50] Chyliński F., Łukowski P. (2016). Management of hazardous waste from the production of titanium dioxide as a substitute for part of cement in cement composites. Mater. Bud..

[51] Ramachandran V.S., Paroli R.M., Beaudoin J.J., Delgado A.H. (2009). The Handbook of Thermal Analysis of Construction Materials.

[52] Han F., Zhang Z., Liu J., Yan P. (2017). Effect of water-to-binder ratio on the hydration kinetics of composite binder containing slag or fly ash. J. Therm. Anal. Calorim..

[53] Alahrache S., Winnefeld F., Champenois J.B., Hesselbarth F., Lothenbach B. (2016). Chemical activation of hybrid binders based on siliceous fly ash and Portland cement. Cem. Concr. Compos..

[54] Ercikdi B., Cihangir F., Kesimal A., Deveci H., Alp Ì. (2010). Effect of natural pozzolans as mineral admixture on the performance of cemented-paste backfill of sulphide-rich tailings. Waste Manag. Res..

[55] Ramakokovhu M.M., Mojisola T., Mbaya R.K.K., Olubambi P.A. An electrochemical study and thermodynamic quantification of pre-oxidized ilmenite metastable phases. Proceedings of the 11th International Heavy Minerals Conference.

[56] Qing Y., Zenan Z., Deyu K., Rongshen C. (2007). Influence of nano-SiO_2_ addition on properties of hardened cement paste as compared with silica fume. Constr. Build. Mater..

[57] Chen Y., Deng Y., Li M. (2016). Influence of Nano-SiO_2_ on the consistency, setting time, early-age strength, and shrinkage of composite cement pastes. Adv. Mater. Sci. Eng..

[58] Indukuri C.S.R., Nerella R., Madduru S.R.C. (2019). Effect of graphene oxide on microstructure and strengthened properties of fly ash and silica fume based cement composites. Constr. Build. Mater..

[59] Rossen J.E., Lothenbach B., Scrivener K.L. (2015). Composition of C-S-H in pastes with increasing levels of silica fume addition. Cem. Concr. Res..

